# Complete genome sequence of *Ornithobacterium rhinotracheale* strain ORT-UMN 88

**DOI:** 10.1186/1944-3277-9-16

**Published:** 2014-12-08

**Authors:** Emilie S Zehr, Darrell O Bayles, William D Boatwright, Louisa B Tabatabai, Karen B Register

**Affiliations:** 1 Ruminant Diseases and Immunology Research Unit, U. S. Department of Agriculture, Agricultural Research Service, National Animal Disease Center, Ames, IA, USA; 2 Infectious Bacterial Diseases Research Unit, U. S. Department of Agriculture, Agricultural Research Service, National Animal Disease Center, Ames, IA, USA; 3 Roy J. Carver Department of Biochemistry, Biophysics and Molecular Biology, Iowa State University, Ames, IA, USA

**Keywords:** *Ornithobacterium rhinotracheale*, Poultry, Respiratory disease, Genome sequence

## Abstract

*Ornithobacterium rhinotracheale* strain ORT-UMN 88 is a Gram-negative, pleomorphic, rod-shaped bacterium and an etiologic agent of pneumonia and airsacculitis in poultry. It is a member of the family *Flavobacteriaceae* of the phylum *Bacteroidetes. O. rhinotracheale* strain ORT-UMN 88 was isolated from the pneumonic lung of a turkey in 1995. It was the isolate first used to experimentally reproduce disease in turkeys and has since been the focus of investigations characterizing potential virulence factors of the bacterium. The genome of *O. rhinotracheale* strain ORT-UMN 88 consists of a circular chromosome of 2,397,867 bp with a total of 2300 protein-coding genes, nine RNA genes, and one noncoding RNA gene. A companion paper in this issue of SIGS reports the non-contiguous finished genome sequence of an additional strain of *O. rhinotracheale*, isolated in 2006.

## Introduction

*Ornithobacterium rhinotracheale* appears to have been the cause of respiratory disease in poultry since at least 1981 [1], although its biochemical characteristics and phylogenetic position were not determined until the early 1990’s [2,3]. It is a global pathogen and has been isolated not only from turkeys and chickens but also a variety of other domesticated and wild birds, including chukar partridges, ducks, geese, guinea fowl, gulls, ostriches, partridges, pheasants, pigeons, quail, rooks, and falcons [4,5]. A typing scheme based on the reaction of monospecific antisera with heat-extracted antigens has so far discriminated 18 serotypes of *O. rhinotracheale*, designated as A through R [1,4], although not all isolates are typeable. The most common clinical signs of disease related to *O. rhinotracheale* are pneumonia, tracheitis, airsacculitis, sinusitis, and pericarditis [1,4]. These infections impose a substantial economic burden on the poultry industry worldwide, due to decreased egg production, reduced eggshell quality and hatchability, reduced weight gain, increased mortality, and increased condemnation rates [6-9]. Whole-cell bacterin and live, attenuated vaccines have met with limited success, likely due to the lack of cross-protection against heterologous serotypes. Recent studies have identified antigens that appear to provide cross-protective immunity when formulated as a recombinant, multi-component subunit vaccine [10].

*O. rhinotracheale* strain ORT-UMN 88 was isolated from the pneumonic lung of a turkey and classified as serotype A at the University of Minnesota, St. Paul, MN in 1995 [11]. This isolate was used as a challenge strain to reproduce the disease in previously healthy turkeys [12]. Further characterization revealed a dependence on iron for maximal growth, acquired through a mechanism apparently unrelated to siderophore production but possibly via an iron-bound protein pathway [13]. Although *O. rhinotracheale* has generally been considered nonhemolytic on blood agar, Tabatabai et al. [14] documented the β-hemolytic activity of numerous strains, including *O. rhinotracheale* strain ORT-UMN 88, and suggested that a hemolysin-like protein may function as a virulence factor. The availability of genome sequence data from *O. rhinotracheale* strains of known virulence will greatly facilitate efforts to define virulence factors and the molecular basis for pathogenesis. Here we present a description of the complete genome of *O. rhinotracheale* strain ORT-UMN 88 and its annotation. This isolate (alias P5887) was provided to the National Animal Disease Center by the University of Minnesota and is available upon request from the National Animal Disease Center Biological Agent Archive and Culture Collection.

## Organism information

### Classification and features

**Figure 1:**
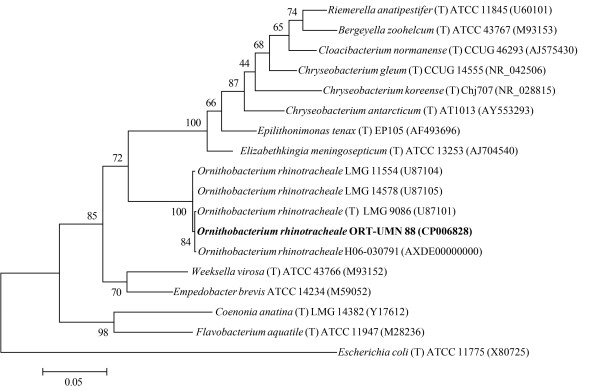
**Phylogenetic tree based on 16S rRNA showing the position of *O. rhinotracheale *ORT-UMN 88 (highlighted in bold) in relation to other *O. rhinotracheale *isolates for which sequence is available and to the type strains (T) of closely related species and genera within the family *Flavobacteriaceae*. ***Escherichia coli* (a member of the *Enterobacteriaceae* family) was included as an outgroup. An internal region of the 16S rRNA (1251 bp with no gap-containing sites) was aligned using CLUSTALW, and phylogenetic inferences were obtained using the maximum likelihood method with the Jukes-Cantor model within MEGA version 5.10 software [16]. Numbers at the nodes are percentages of bootstrap values obtained by repeating the analysis 1000 times to generate a majority consensus tree. GenBank accession numbers for the sequences are given in parentheses. The scale bar represents 5% substitution per nucleotide position.

The genus *Ornithobacterium* belongs to the class “*Flavobacteriia"* and is in the family *Flavobacteriaceae*[15] (Table 1). *O. rhinotracheale* is the sole species within the genus. Phylogenetic analysis based on 16S ribosomal RNA of *O. rhinotracheale* and other genera within the *Flavobacteriaceae* family is shown in Figure 1. The 16S rRNA sequences of *O. rhinotracheale* strain ORT-UMN 88 and the type strain, LMG 9086, share 99.9% nucleotide sequence identity. Three rRNA loci were found in the *O. rhinotracheale* strain ORT-UMN 88 genome. All *O. rhinotracheale* strains in Figure 1 were isolated from turkeys, with the exception of strain LMG 11554, which was cultured from a rook.

**Table 1:** **Classification and general features of *O. rhinotracheale *** **strain ORT-UMN 88 in accordance with the MIGS recommendations**[17]

**MIGS ID**	**Property**	**Term**	**Evidence code**^a^
	Current classification	Domain “*Bacteria”*	TAS [18,19]
		Phylum “*Bacteroidetes”*	TAS [20,21]
		Class “*Flavobacteriia”*	TAS [22,23]
		Order *Flavobacteriales*	TAS [24,25]
		Family *Flavobacteriaceae*	TAS [15,26,27]
		Genus *Ornithobacterium*	TAS [28,29]
		Species *rhinotracheale*	TAS [28,29]
MIGS-7	Subspecific genetic lineage (strain)	Strain ORT-UMN 88	TAS [13]
		Serotype A	TAS [13]
	Gram stain	Negative	TAS [1,4]
	Cell shape	Pleomorphic rod	TAS [1,4]
	Motility	Nonmotile	TAS [1,4]
	Sporulation	Non-sporulating	TAS [1,4]
	Temperature range	Mesophile (30°C-42°C)	TAS [1,4]
	Optimum temperature	37°C	TAS [1,4]
MIGS-6.2	pH range; Optimum	7.2-7.6 (BHI); 7.4	TAS [1], IDA
	Carbon source	Saccharolytic (glucose)	TAS [4]
MIGS-6	Habitat	Respiratory tract of birds worldwide	TAS [1,4]
MIGS-6.3	Salinity	Growth in BHI broth, (0.75% salts)	TAS [1], IDA
MIGS-22	Oxygen requirement	Microaerophilic, anaerobic, or aerobic	TAS [1,4]
	Energy metabolism	Chemoorganotroph	TAS [4]
MIGS-15	Biotic relationship	Parasitic	TAS [4]
MIGS-14	Pathogenicity	Pneumonia, airsacculitis, tracheitis, pericarditis	TAS [1,12]
MIGS-16	Specific host	Poultry	TAS [1,4,12]
MIGS-18	Health status of host	Symptomatic	TAS [12,13]
	Biosafety level	2 t	TAS [30]
MIGS-19	Trophic level	Chemoheterotroph	TAS [4]
MIGS-23.1	Isolation	Pneumonic turkey lung	TAS [12,13]
MIGS-4	Geographic location	Minnesota, USA	TAS [12,13]
MIGS-5	Time of sample collection	1995	TAS [12]
MIGS-4.1	Latitude	Not reported	
MIGS-4.2	Longitude	Not reported	
MIGS-4.3	Depth	Not reported	
MIGS-4.4	Altitude	Not reported	

^a^Evidence codes - IDA: Inferred from Direct Assay; TAS: Traceable Author Statement (i.e., a direct report exists in the literature); NAS: Non-traceable Author Statement (i.e., not directly observed for the living, isolated sample, but based on a generally accepted property for the species, or anecdotal evidence). Evidence codes are from the Gene Ontology project [31].

**Figure 2:**
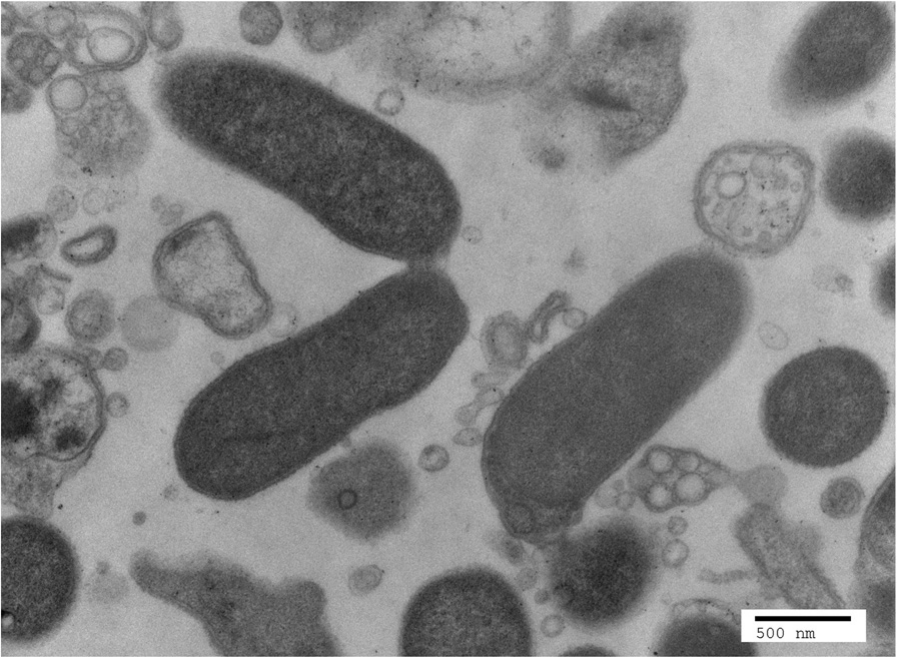
**Transmission electron micrograph of *O. rhinotracheale *strain ORT-UMN 88 cells cultured in broth, using a Tecnai G**^**2 **^**(FEI, Hillsboro, OR) at an operating voltage of 80 kV.** The average length of representative cells was 1.7 μm and the average width was 0.64 μm. The scale bar represents 500 nm.

*O. rhinotracheale* strain ORT-UMN 88 cells grown in broth medium are Gram-negative, pleomorphic rods, ranging from 1.53-1.86 μm (mean, 1.70 μm) in length compared to 0.59-0.72 μm (mean, 0.64 μm) in width (Figure 2). The bacterium is nonmotile and microaerophilic, preferring 7.5% CO_2_ humidified atmosphere from 30°C to 42°C for growth. Colonies are approximately 1 mm in diameter and yellowish in color after 48 h incubation at 37°C on blood agar. Unlike type strain LMG 9086 [3], *O. rhinotracheale* strain ORT-UMN 88 is β-hemolytic on 5% sheep blood agar [14].

Results of biochemical tests for *O. rhinotracheale* strains can be variable and strain-dependent [1]. After seven days of incubation at 37°C, *O. rhinotracheale* strain ORT-UMN 88 is weakly acidic on a triple sugar iron agar slant and does not produce hydrogen sulfide or gas. Lactose and dextrose are weakly fermented and galactose, sucrose, sorbitol, xylose, and mannitol are not fermented, with or without the addition of 2% chicken serum. The isolate is lysine decarboxylase positive, ornithine decarboxylase negative, and urease positive.

### Genome sequencing information

#### Genome project history

*O. rhinotracheale* strain ORT-UMN 88 was selected for sequencing on the basis of prior *in vivo* and *in vitro* characterization [12-14]. The Whole Genome Shotgun project and complete genome sequence of *O. rhinotracheale* strain ORT-UMN 88 has been deposited at DDBJ/EMBL/GenBank under the accession no. CP006828. Sequencing, finishing, and final annotation were performed at the DNA Facility of Iowa State University and the National Animal Disease Center, Ames, IA. A summary of the project information is given in Table 2.

**Table 2:** **Project information of *O. rhinotracheale *** **strain ORT-UMN 88**

**MIGS ID**	**Property**	**Term**
MIG-31	Finishing quality	Finished
MIGS-28	Libraries used	Three genomic libraries: two shotgun libraries, one mate-pair (8 kb insert size) library
MIGS-29	Sequencing platforms	Illumina GA II, Roche GS FLX Titanium, Sanger
MIGS-31.2	Fold coverage	61x (36x Roche FLX, 26x Illumina); final SEQuel error correction with 100x Illumina
MIGS-30	Assemblers	MIRA v3.4.0, Roche gsAssembler v2.8
MIGS-32	Gene calling method	GeneMarkS + (NCBI PGAP)
	GenBank ID	CP006828
	GenBank Date of Release	September 22, 2014
	GOLD ID	Gi0071044
	NCBI project ID	219465
	Project relevance	Poultry respiratory pathogen
MIGS-13	Source material identifier	ORT-UMN 88

### Growth conditions and genomic DNA preparation

A clonal population of *O. rhinotracheale* strain ORT-UMN 88, derived from three serial passages of a single colony, was archived at -80°C for future analysis. The bacterium was grown on 5% sheep blood agar plates (Becton, Dickinson and Company, Sparks, MD) incubated for 48 h at 37°C with 7.5% CO_2_ and 15% humidity. Colonies were used to inoculate 5 ml of brain heart infusion broth in a snap-cap tube which was incubated at 37°C for 24 h with rotation at 100 rpm. Ten ml of these 24 h cultures were then diluted 10-fold into fresh BHI broth. The newly inoculated culture was incubated in a 250-ml flask at 37°C for 48 h at 75 rpm (final OD_600_ = 0.371). An aliquot was plated on 5% sheep blood agar to confirm purity and 10 ml was removed for DNA preparation. Cells were pelleted successively into one 2-ml microfuge tube at 16,000 × g. Genomic DNA was isolated using the Wizard Genomic DNA Purification Kit (Promega Corporation, Madison, WI) with the following modifications: the cell pellet was resuspended in 480 μl of 200 mM EDTA, 60 μl of 10 mg/ml lysozyme, and 60 μl of double distilled water before lysis, then 10 μl of 10 mg/ml RNase solution was added to the cell lysate. The precipitated genomic DNA was rehydrated at 65°C for 1 h in 10 mM Tris-HCl, pH 8.5, evaluated on a 6% agarose gel to verify the lack of low molecular weight fragments, and quantified with Quant-iT PicoGreen dsDNA Assay Kit (Invitrogen, Carlsbad, CA).

### Genome sequencing and assembly

A fully scaffolded genome was assembled using MIRA v. 3.4 [32] and the Roche gAssembler v. 2.6 to achieve 61 × total genome coverage through the assembly of Roche GS FLX shotgun, GS FLX large insert (7.9 kb) mate pair, Illumina 75-bp single direction, and Illumina 2 × 75 bp paired-end sequencing reads. All remaining sequencing gaps in the scaffolded assembly were PCR amplified and sequenced by the Sanger method. GAP5 [33], from the Staden Package, was used as the editor for incorporating the gap-closing sequences, ultimately resulted in a completely closed genome. Base calling errors in the closed genome assembly were corrected by using SEQuel [34] to map Illumina reads back to the genome at approximately 100 × total genome coverage.

### Genome annotation

The assembled genome was submitted to the National Center for Biotechnology Information (Bethesda, MD) through the Whole Genome Shotgun genome sequencing portal [35] and genes were identified using the NCBI Prokaryotic Genome Annotation Pipeline. Signal peptides were identified with the SignalP 4.0 software [36], transmembrane helices were classified with the method of Krogh et al. [37], and the CRISPR motif was detected with a web tool described by Griss et al. [38].

## Genome properties

**Figure 3:**
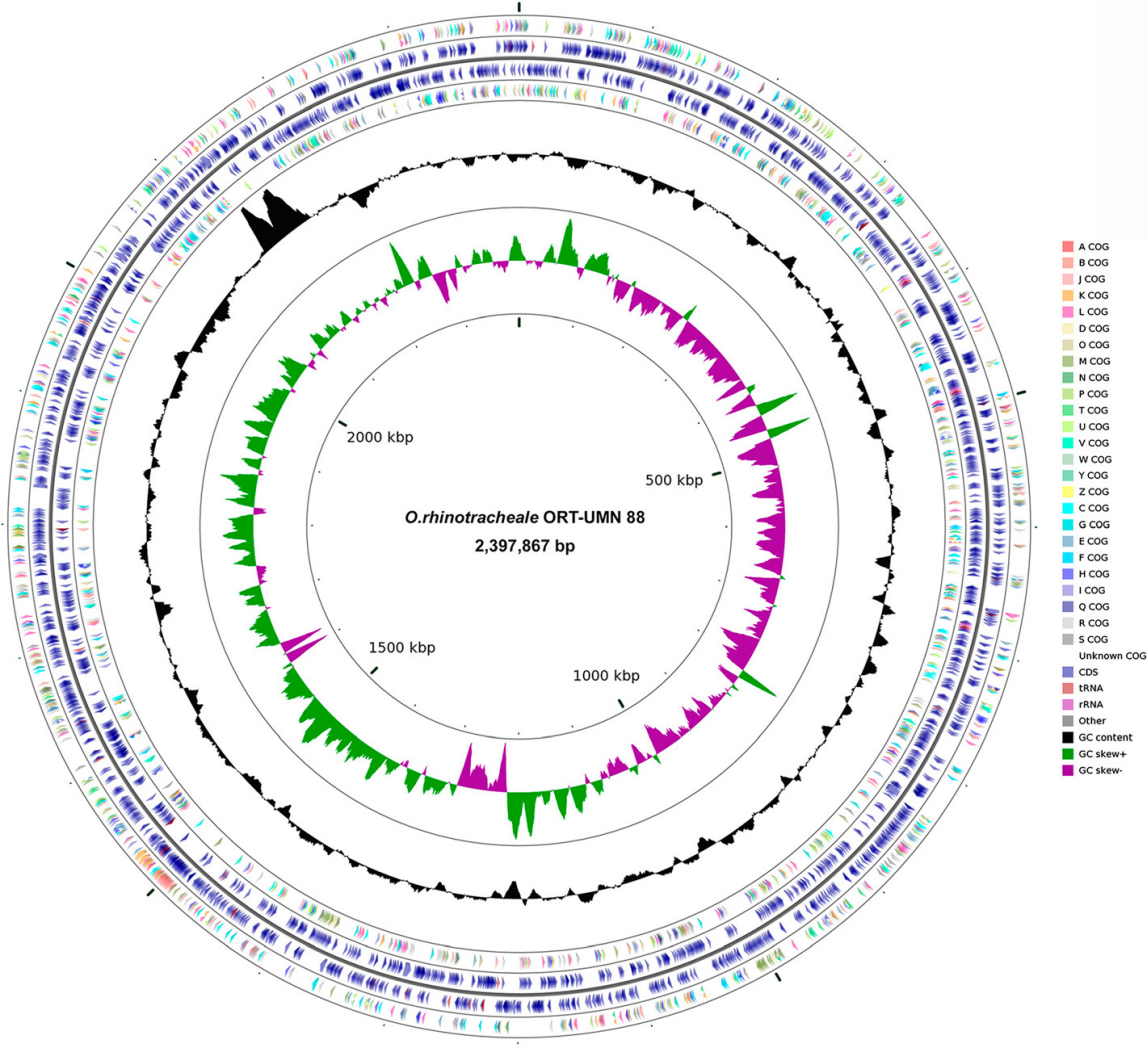
**Graphical map of the *O. rhinotracheale *strain ORT-UMN 88 chromosome.** From outside to the center: genes on forward strand (color by COG categories), CDS on forward strand, tRNA, rRNA, other; CDS on reverse strand, tRNA, rRNA, other, genes on reverse strand (color by COG categories); GC content; GC skew, where green indicates positive values and magenta indicates negative values.

The genome properties and statistics of *O. rhinotracheale* strain ORT-UMN 88 (Accession CP006828) are shown in Tables 3 and 4 and Figure 3. The complete genome consists of one circular 2,397,867 bp chromosome with a 34.22% G + C content and no plasmids. Of the 2,389 genes predicted, 2,300 are protein-coding genes, 36 are pseudogenes, nine are RNA genes, and one is a noncoding RNA gene. The majority (59.61%) of the protein-coding genes were assigned a putative function. The distribution of genes into COGs functional categories is presented in Table 4. Additionally, one CRISPR motif was detected.

**Table 3:** **Genome statistics of *O. rhinotracheale *** **strain ORT-UMN 88**

**Attribute**	**Genome (total)**	
	**Value**	**% of total**^**b**^
Genome size (bp)	2,397,867	100.00%
DNA coding (bp)	2,138,862	89.20%
DNA G + C (bp)	820,557	34.22%
Total genes^a^	2389	100.00%
Protein-coding genes	2300	93.89%
RNA genes	9	3.77%
rRNA operons	3	
tRNA genes	43	1.80%
Pseudo genes	36	1.60%
Genes with function prediction	1337	59.61%
Genes assigned to COGs	1374	61.26%
Genes assigned Pfam domains	1494	66.61%
Genes with signal peptides	270	12.04%
Genes with transmembrane helices	500	22.29%
CRISPR repeats	1	

^a^Total genes include one noncoding RNA gene

^b^The total is based on either the size of the genome in base pairs or the total number of protein coding genes in the annotated genome.

**Table 4:** **Number of genes associated with the 25 general COG functional categories of *O. rhinotracheale *** **strain ORT-UMN 88**

**Code**	**Value**	**% age**^**a**^	**Description**
J	133	5.57	Translation, ribosomal structure and biogenesis
A	0	0	RNA processing and modification
K	47	1.97	Transcription
L	116	4.86	Replication, recombination and repair
B	0	0	Chromatin structure and dynamics
D	20	0.84	Cell cycle control, cell division, chromosome partitioning
Y	0	0	Nuclear structure
V	34	1.42	Defense mechanisms
T	26	1.09	Signal transduction mechanisms
M	118	4.94	Cell wall/membrane biogenesis
N	3	0.13	Cell motility
Z	0	0	Cytoskeleton
W	0	0	Extracellular structures
U	29	1.21	Intracellular trafficking and secretion
O	66	2.76	Posttranslational modification, protein turnover, chaperones
C	74	3.1	Energy production and conversion
G	75	3.14	Carbohydrate transport and metabolism
E	109	4.56	Amino acid transport and metabolism
F	52	2.18	Nucleotide transport and metabolism
H	91	3.81	Coenzyme transport and metabolism
I	42	1.76	Lipid transport and metabolism
P	79	3.31	Inorganic ion transport and metabolism
Q	17	0.71	Secondary metabolites biosynthesis, transport and catabolism
R	153	6.4	General function prediction only
S	90	3.77	Function unknown

^a^The total is based on the total number of protein coding genes in the annotated genome.

## Conclusions

Other than the genome sequence of *O. rhinotracheale* strain ORT-UMN 88, reported here, genome sequences are available from only two additional isolates of the bacterium. The type strain, LMG 9086, was the first to be sequenced to completion. Those data were made publically available in GenBank in 2012, but a corresponding analysis has yet to be reported in the scientific literature. A non-contiguous finished genome sequence comprising seven contigs from strain H06-030791 is reported in a companion paper in this issue of SIGS [39].

Comparison of the aligned genomes of LMG 9086 and ORT-UMN 88 revealed that large rearrangements and inversions are the prominent distinguishing features. Relative to the type strain, the genome of ORT-UMN 88 contains a single inverted region of ~18 Kb and two regions that are both inverted and rearranged, one of ~354 Kb and the other of ~228 Kb, each with a transposase or transposon found at one terminus. These same regions are similarly inverted and rearranged in the genome of strain H06-030791. However, H06-030791 has an additional rearrangement of ~33 Kb and an inverted and translocated stretch of ~59 Kb, neither of which is similarly altered in ORT-UMN 88. Numerous smaller rearrangements can also be found in the sequence of ORT-UMN 88 relative to that of LMG 9086. As one example, in a region otherwise syntenous, homologs for 12/14 CDSs of ORT-UMN 88 (locus tags Q785_00290-00360) are absent from this region of LMG 9086 and are instead located roughly 359 Kb distant. At the point of divergence in LMG 9086 is a CDS encoding a membrane-associated phosphatase for which no homolog is evident in ORT-UMN 88. Transposases border the regions of rearrangement in both isolates. Homologs for five of the 14 ORT-UMN 88 CDSs mentioned above are found at the same position in the ORT-UMN 88 and H06-030791 genomes. H06-030791 homologs of the remaining nine CDSs are split between two different regions, both distantly located, neither of which corresponds to the region in which they are found in LMG 9086. The LMG 9086 membrane-associated phosphatase noted above to be absent from ORT-UMN 88 is also not apparent in the H06-030791 genome. These rearrangements, and several others examined, are nearly always bordered by transposons, transposases or insertion sequences and are often accompanied by duplication of one or a few CDSs present at the points of sequence divergence. An additional prominent feature of the ORT-UMN 88 genome is an insertion of ~47 Kb comprising 45 CDSs (locus tags Q785_10465-10695), not present in either LMG 9086 or H06-030791, identified as an integrative and conjugative element using a web-based ICE resource [40]. ICEs are self-transmissible elements found in some Gram-positive and Gram-negative bacteria that often confer new phenotypes upon the recipient due to co-transfer of antibiotic resistance genes, virulence factors and other traits [41]. The ORT-UMN 88 ICE contains the protypical integration/excision, conjugation and regulation modules as well as a tetracycline resistance element, a chemotaxis protein gene and a histidine kinase gene. Thus, it appears that multiple classes of mobile elements likely play a role in shaping the genome structure and evolution of *O. rhinotracheale*.

While there are major organizational differences among the genomes of all three isolates, ORT-UMN 88 and H06-030791 share a higher degree of synteny with one another than either shares with LMG 9086. An additional feature shared exclusively between ORT-UMN 88 and H06-030791 is a deletion in both of ~37 Kb found in LMG 9086, comprised primarily of CDSs annotated as hypothetical proteins but also including a holin family protein, an ATP-dependent serine protease, a helix-turn-helix protein and several phage-related proteins. A number of CDSs in ORT-UMN 88 with obvious sequence divergence as compared to homologs in LMG 9086 are identical or nearly identical to homologs in H06-030791 [39]. These include several annotated as hypothetical proteins, an ROK family transcriptional regulator/sugar kinase, a Crp/Fnr family transcriptional regulator and the integral membrane protein and ferrous iron transporter FeoB. Both ORT-UMN 88 and H06-030791 were isolated in the United States, in 1995 and 2006, respectively, while LMG 9086 was isolated in United Kingdom in 1994, suggesting that distinct clones of *O. rhinotracheale* may circulate in geographically distant locales.

ORT-UMN 88 is unique from the type strain with regard to its β-hemolytic phenotype when grown under appropriate conditions on blood agar [14]. A search of the ORT-UMN 88 genome for CDSs whose annotations suggest a function in hemolytic activity revealed only three candidates. Identical or nearly identical homologs were found in the LMG 9086 genome as well as in the genome of the β-hemolytic isolate H06-030791 [39]. An additional CDS annotated in LMG 9086 as a hemolysin was also found in both ORT-UMN 88 and H06-030791, with one and zero amino acid substitutions, respectively, relative to the type strain. Dependence on iron for growth *in vitro* is another phenotype known to be variable among the three *O. rhinotracheale* strains sequenced, with only H06-030791 capable of vigorous growth in the presence of an iron chelator [13]. Among ~30 CDSs collectively found in all three genomes whose annotations suggest a role in iron acquisition or transport, all but FeoB are highly conserved among all isolates. As noted above, FeoB is predicted to be identical in ORT-UMN 88 and H06-030791 but unique in LMG 9086. All three isolates share 100% amino acid identity over the N-terminal 395 amino acids but LMG 9086 has only 94.7% amino acid identity with ORT-UMN 88 and H06-030791 over the C-terminal 301 amino acids.

The genome sequence of ORT-UMN 88 reported here provides additional insights into the genetic structure and evolution of *O. rhinotracheale*. Additional analysis and experimentation is needed to understand the genetic basis of virulence in this bacterium but the availability of genome sequences from three genetically distinct isolates will greatly facilitate related efforts.

## Competing interests

The authors declare that they have no competing interests.

## Authors’ contributions

EZ participated in genome sequencing and drafted the original manuscript. DB directed genome sequence assembly and bioinformatics analyses. WB participated in genome sequencing and post-sequencing analyses. LT conceived of the study and participated in genome sequencing. KR participated in post-sequencing analysis and revised the manuscript. All authors read and approved the final manuscript.
